# The Role of microRNA in the Regulation of Differentiation and the Functionality of Osteoblasts, Osteoclasts, and Their Precursors in Osteoporosis

**DOI:** 10.3390/ncrna11010014

**Published:** 2025-02-08

**Authors:** Bulat I. Yalaev, Elena I. Kaletnik, Yulia S. Karpova, Zhanna E. Belaya, Ildar R. Minniakhmetov, Natalia G. Mokrysheva, Rita I. Khusainova

**Affiliations:** Endocrinology Research Centre, Dmitry Ulyanov Street, 11, 117292 Moscow, Russia; helen2000hkalietnik@gmail.com (E.I.K.); karpova.yuliya@endocrincentr.ru (Y.S.K.); belaya.zhanna@endocrincentr.ru (Z.E.B.); minniakhmetov.ildar@endocrincentr.ru (I.R.M.); mokrisheva.natalia@endocrincentr.ru (N.G.M.); khusainova.rita@endocrincentr.ru (R.I.K.)

**Keywords:** osteoporosis, epigenetics, microRNAs, differentiation, osteoblasts, osteoclasts, bone, precursors, tissue, regulation, diagnostics, therapy

## Abstract

Osteoporosis is a complex disease that is affected by a variety of factors, including genetic and epigenetic influences. While DNA markers for osteoporosis have been identified, they do not fully explain the hereditary basis of the disease. Epigenetic factors, such as small microRNAs (miRNAs), may provide a missing link in understanding the molecular mechanisms underlying osteoporosis. miRNAs are a class of non-coding RNAs that play a role in the epigenetic regulation of gene expression. They are known to be involved in various biological processes, including bone formation and remodelling. Differential expression of miRNAs has been linked to the pathological decrease in bone mineral density associated with osteoporosis. It has been shown that an abnormal miRNA expression pattern leads to a decrease in osteoblast activity and an increase in osteoclast activity. Further research into the role of miRNAs in osteoporosis may help to better understand this disease and identify potential therapeutic targets for treatment. Based on these assumptions, the study of miRNA expression patterns in osteoblasts, osteoclasts, and their precursors under normal and osteoporotic conditions is a rapidly growing field of scientific research. Although the results of this research are still incomplete and sometimes contradictory, they require additional scientific analysis to better understand the complex mechanisms involved. The purpose of this paper is to review the current research on miRNAs specifically expressed in osteoblasts and osteoclasts under both normal and pathological conditions. We will also discuss the potential applications of these miRNAs as biomarkers for osteoporosis diagnosis and as targets for osteoporosis treatment.

## 1. Introduction

Osteoporosis (OP) is one of the most common multifactorial diseases leading to bone fragility and a high incidence of low traumatic fractures in adults. The disease is socially significant, as it is associated with disability and high mortality after fracture due to the latent course of the disease and low early detection rates. According to a meta-analysis, the global prevalence of osteoporosis is more than 18% worldwide [1]. The disease is diagnosed in 34% of women and 27% of men in densitometric examinations in independent or random samples in Russia [2,3]. Injured people experience a significant deterioration in the quality of life, and mortality increases dramatically. The key solution to these problems is the development of precision medicine aimed at preventing fractures based on early diagnosis of OP. A significant place in the early diagnosis of osteoporosis is occupied by fundamental research into the molecular mechanisms that contribute to increased fracture risk and the formation of low bone mineral density (BMD). The development of primary osteoporosis is influenced by pathogenic changes in both coding and non-coding DNA sequences that have been studied so far, excluding factors such as dietary patterns, lifestyle, and other risk factors. Research on epigenetic abnormalities related to osteoporosis is still in its early stages [4,5].

It is known that key epigenetic changes in osteoporosis affect the regulation of signalling pathways involved in mesenchymal stem cell (MSC) differentiation, including the activity of the transcription factor RUNX2, sclerostin, and DKK1 [5]. Additionally, the RANKL-RANK-OPG system is affected by genetic alterations [6] and epigenetic aberrations [7]. Differential DNA methylation and changes in the expression of non-coding RNA in osteoporosis lead to alterations in the activity of WNT signalling, osteoclast differentiation, and variable transcription factors. Thus, epigenetic factors can affect the levels of various biologically active substances that regulate the bone remodelling process [8].

The posttranslational modification of histones plays a crucial role in the formation of bone structure and its mineralization. In osteoporosis, there is a reduction in the acetylation of the H3K9/K14 and H4K12 regions in the regulatory regions of the *RUNX2* and *OSX* genes, and an increase in acetylation at these same sites in the regulatory region of the *PPARy2* gene in mesenchymal stem cells (MSCs) derived from bone marrow. The *HOXA10* gene promotes osteogenic determination by enhancing the trimethylation of the fourth lysine residue on histone, which activates RUNX2, alkaline phosphatase, and osteocalcin, stimulating bone cell maturation. The transcriptional activity of the *RUNX2* gene is enhanced by the action of acetyltransferase P300 and nicotinamide-phosphoribosyltransferase (NAMPT), which in turn promotes the osteogenic differentiation of MSCs through the acetylation of H3K14 in MC3T3-E1 cells and H3K9 in MSCs, respectively [9].

It was found that the activity of genes *MEPE*, *SOST*, *WIF1*, and *DKK1*, which are metabolic inhibitors of bone tissue formation, correlated strongly with methylation at a large number of CpG sites in different genes. In addition, some methylated CpG sites were identified, and the level of these sites differed significantly between osteoporotic patients and a control group. The highest level of significance was observed for the *RAD23* homologous B gene (eg14919562), the *PEX14* gene (peroxisomal biogenesis factor 14) (eg14170597), the *TN-X* gene (tenascin XB) (cg03822479), etc. A decrease in methylation levels in the *RAD23* gene was seen in women with osteoporosis, while methylation levels were increased in four women [10]. A full-genomic analysis in women with early postmenopausal osteoporosis revealed differences in methylation patterns among the *ZNF267*, *ABLIM2*, *RHOJ*, *CDKL5*, *PDCD1*, *ABRA*, and *HOJ* genes [11]. Currently, most research and data on the epigenetics of osteoporosis focuses on miRNAs. Studies have shown that changes in miRNA expression are associated with an increased risk of fractures and lower bone mineral density [12].

Two major scientific issues have come to light after several decades of studying the epigenetic basis of primary osteoporosis [9,13]:

1. Disruptions in RNA interference, histone modifications, and DNA methylation affect the risk of disease development. However, epigenetic mechanisms are dynamic and reversible, and the identification of their specific patterns at different disease stages is a non-trivial task.
2. OP is a heterogeneous disease with different etiopathogenesis. Multiple factors influence the risk of the disease, which together complicate the search for causative factors of OP among epigenetic mechanisms.

Osteoblasts and osteoclasts are responsible for the formation and maintenance of bone tissue homeostasis. The coordinated and balanced functioning of these cells affects bone remodelling and the natural process of bone formation and resorption. In turn, the functional activity of these cells depends on the complex regulation of their progenitor cells [14]. The precursors of osteoblasts are mesenchymal stem cells [15], and osteoclasts are mononuclear cells from the hematopoietic lineage, which originates from multipotent myeloid stem cells [16]. Mononuclear progenitor cells of the immature macrophage lineage contain a subset of specifically expressed miRNAs, which are associated with the regulation of the differentiation of these cells into osteoclasts. This is also true for specific miRNA patterns in MSCs differentiating into osteoblasts. Disturbance to miRNA expression regulation may be associated with a pathological enhancement of the proliferation induction and resorption functions of bone cells in OP [17]. However, the aberrant expression profile of miRNAs has not been studied sufficiently to create a miRNA atlas and investigate the interactome of miRNAs and mRNAs in OP. This would be a turning point in the development of advanced solutions for early diagnosis of and therapy for the disease.

## 2. General Information on the Role of Non-Coding RNAs in Bone Biology

The search for DNA markers (such as single nucleotide polymorphism (SNP), etc.) of OP has helped to identify only a fraction of the molecular genetic predictors of the disease. Family, sibship and twin studies have shown that OP-associated changes in DNA explain only 60–80% of the variability in bone mineral density (BMD) levels and only up to 50–70% of the risk of osteoporotic fractures [18]. Therefore, it can be assumed that the remaining percentage of undiscovered risk factors for osteoporosis in general, as well as for fractures and low BMD in particular, is most likely attributable to epigenetic mechanisms, including changes in the activity of regulatory non-coding RNAs (ncRNAs). Studies have identified long non-coding RNAs (lncRNAs) (more than 200 nucleotides in length) and circular RNAs that are involved in bone formation and remodelling. As it turned out, lncRNAs play a prominent role in the differentiation of osteoblasts [19,20]. For example, Yuan et al. found that expression of the long non-coding RNA PGC1β-OT1, which stands for peroxisome proliferator-activated receptor γ coactivator, enhances osteogenic differentiation [21]. Circular RNAs are directly involved in the regulation of osteoclast differentiation [22]. The level of the circular RNA with the identifier ‘007438’ is elevated during osteoclastogenesis. It targets miRNA-6338 and miRNA-7028-3p; while the circular RNA numbered ‘005108’ is decreased during osteoclastogenesis, its targets are miRNA-6975-3p, miRNA-6516-5p, miRNA-486b-5p, and miRNA-31-3p [23]. Сircular and lncRNAs can act as miRNA sponges by inhibiting their activity on target mRNAs [24].

miRNAs are considered the most studied, not only among non-coding RNAs in general, but also among all epigenetic factors that influence the risk of OP [5]. These molecules are approximately 20∼25 nucleotides long and represent a class of evolutionarily conserved non-coding small RNA molecules. There are around 2600 mature miRNAs in the human genome, which control the activity of more than 60% of protein-coding genes [25]. Together with small interfering RNAs or separately, miRNAs are involved in the process of RNA interference—a system of gene expression suppression (silencing) at the level of the transcription, translation, deadenylation, or degradation of mRNA [26].

Differential expression of miRNAs controls key factors in cell maturation and proliferation involving transforming growth factor-β (TGF-β), bone morphogenetic protein (BMP), WNT and NOTCH signalling pathways, RUNX2 transcription factor, OSTERIX (OSX), and DLX5, which play a direct role in osteoblast maturation [27,28]. miRNAs have been conclusively shown to play critical roles in fracture healing and osteoblast function. Specifically, miR-21 is associated with improved mineralisation during osteogenic induction, while miR-16-5p, in contrast, inhibits this process [29]. Ghayor and Weber, in their study, identified two groups of miRNAs that promote either differentiation or lead to the inhibition of osteoclast and osteoblast maturation. They found that miR-216-a, miR-21, miR-194, and miR-96 are effectors (i.e., promoting maturation) of osteoblasts, and miR-23-a, miR-375, miR-153, and miR-124 are their inhibitors, respectively. In the second group, the authors included miR-214, miR-183, miR-97, and miR-18, which are inducers of osteoclasts, and, in contrast, miR-1720a, miR-26-a, miR-7-b, miR34-a, and miR-126-5p are their negative regulators [30]. Chen et al. found that an overexpression of miR-503 targeting RANK in CD14+ cells inhibits osteoclastogenesis. In women with postmenopausal OP, baseline miR-503 levels were lower in monocytes compared to a control sample, thus suggesting an antagonism between miR-503 and bone resorption [31]. Notably, miR-221 inhibits *RUNX2* gene activity in oestrogen hormone deficiency. miR-21 and miR-124a regulate the *PDCD4* and *NFATC1* genes, respectively, and have opposite effects in the RANKL-RANK-OPG system of bone remodelling [32].

The Russian Endocrinology Research Center studied changes in miRNA expression in the bone tissue of patients with some secondary forms of OP [33]. Thus, in endogenous hypercorticism, patients develop severe OP with multiple fractures due to the suppression of osteoblastogenesis due to an upregulation of canonical WNT signalling antagonists and changes in the levels of miR-125b-5p, miR-218-5p, miR-34a-5p, miR-188-3p, and miR-199a-5p, which are associated with the competitive differentiation of MSCs towards adipocytes against osteoblasts [34]. Growth hormone excess in acromegaly patients caused increased expression of WNT signalling antagonists and changes in the levels of miRNAs involved in MSC differentiation towards chondrocytes (miR-199a-5p) or adipocytes (miR-27-5p, miR-125b-5p, miR-34a-5p, miR-188-3p). Compensatory mechanisms have also been identified in patients with acromegaly through increased expression of miRNAs involved in osteoblastogenesis (miR-210-5p, miR-135a-5p, miR-211, miR-23a-3p, miR-204-5p) [34].

Osteocytes are the last level of osteoblast differentiation. Their role is to stabilise the organic and mineral composition of bone, as well as metabolism [35]. Osteocytes secrete extracellular vesicles containing miRNAs that can influence muscle and adipose tissue function [36]. miRNAs whose expression is upregulated during late osteocyte differentiation inhibit the activity of genes required for osteoblast differentiation; among them, miR-101a, miR-10a, and the let-7 and miR-30 are particularly notable [37]. Thus, miRNAs stimulate or inhibit osteoblast differentiation by regulating many genes [38].

## 3. Role of microRNAs in the Regulation of Osteoblast Differentiation and Activity

Osteoblasts are single-nucleated cells lining the inner surface of bone trabeculae, the surface of haversian canals, and the inner layer of periosteum. These cells are the precursors of osteocytes and act as a single system to form a unit of bone tissue known as an osteon. Osteoblasts are involved in the creation of bone matrix and secrete many osteogenic factors such as osteopontin, osteocalcin, and alkaline phosphatase. The process of osteoblastogenesis is divided into three main stages: osteoprogenitor proliferation, preosteoblast maturation, and finally the osteoblastic phase, in which osteoblasts are involved in matrix mineralisation [39]. Up until adolescence, osteoblasts tend to differentiate from the chondrocyte lineage, whereas in adults they predominantly originate from MSCs [40].

The key regulators of osteoblastogenesis are the transcription factors RUNX2 and SP7 (Osterix), and DLX5, WNT, NOTCH, TGFβ, and PI3K/AKT signalling systems, which induce MSC differentiation along the osteogenic pathway [41,42]. The activity of all these factors is closely related to epigenetic regulation. For example, miR-433-3p reduces the expression of the *DKK1* gene (Dickkopf-1), which is an antagonist of the WNT signalling pathway, thereby exerting anabolic effects on bone formation [43]. Duan et al. showed that the upregulation of miR-16-2*, which has an affinity for *WNT5A* gene mRNA, is associated with impaired bone metabolism through decreased *RUNX2* gene activity [44]. miR-148a-3p has been found to enhance both osteoclastogenesis [45] and adipogenesis in osteogenic progenitor cells [46]. The levels of this miRNA in plasma are significantly higher in patients with OP compared to controls without OP [47].

Makitie et al. analysed the levels of 192 circulating miRNAs in patients with autosomal dominant osteoporosis from two Finnish families with a genetically determined WNT signalling disorder, caused by a heterozygous c.652T > G (p.C218G) mutation in exon 4 of the *WNT1* gene. They found that the levels of miR-22-3p, miR-31-5p, miR-34a-5p, miR-143-5p, miR-423-5p, and miR-423-3p were significantly lower in the carriers of the mutation compared to the control group without the mutation. According to the results, these miRNAs are part of the epigenetic regulation of *WNT1* gene expression and can serve as biomarkers for osteoporosis and its treatment. WNT signalling is known to activate osteoblast progenitor cells and promote their differentiation into osteoblasts through a canonical β-catenin-dependent pathway. Based on the available evidence, it can be concluded that miR-22-3p, miR-31-5p, miR-34a-5p, miR-143-5p, and miR-423-5p, as well as miR-423-3p, play a direct role in the epigenetic regulation of osteoblastogenesis, and may be associated with osteoporosis. The effect of these miRNAs depends on the specific mutation in the *WNT1* gene [48]. Thus, the *WNT1* gene variant c. 652 T > G disrupts feedback regulation between these miRNAs and WNT1, representing a particular example of linking genetic polymorphisms to epigenetic disorders in primary OP. Non-canonical WNT signalling receptor FZD3, together with the closely related FZD6, form a distinct subgroup within the Frizzled family. Weilner et al. determined that in plasma from elderly patients, miR-31 is associated with the suppression of MSC differentiation into osteoblasts [49]. Mizoguchi et al. found that stimulation of RANKL in macrophages leads to an 18-fold increase in miR-31 expression levels, and its inhibition by antagomirs leads to increased bone resorption [50].

In 2018, the research group of Ramirez-Salazar et al. published the results of a study on the expression of 754 miRNAs in postmenopausal women with low BMD and osteoporotic fractures compared to control women. The authors concluded that miR-23b-3p and miR-140-3p were significantly associated with osteoporosis in the study sample. Furthermore, in silico functional analysis confirmed the role of miR-140-3p and miR-23b-3p in osteoblast differentiation. Bioinformatic modelling has allowed us to determine that both miR-140-3p and miR-23b-3p regulate signalling pathways involved in WNT, MAPK, and TGF-β, playing a role in osteoblastogenesis activation. At the same time, miR-23b-3p levels are elevated in the serum of patients with low bone mineral density, and miR-140-3p overexpression is observed during fracture healing and in exosomes derived from osteoblasts. miR-140-3p inhibits osteoblast formation by suppressing *BMP2* gene expression [51].

The miR-29 miRNAs include miR-29a, miR-29b, and miR-29c. Based on the prediction of potential binding sites, members of this family may regulate the expression of more than 6000 genes [52]. It is suggested that miR-29 is simultaneously involved in both the regulation of bone remodelling and collagen synthesis, affecting the risk of osteoporosis. This relationship has been repeatedly studied in various studies: miR-29 levels are significantly reduced in the bone tissue of patients with osteoporosis; moreover, a correlation between the expression level of this miRNA and the dynamics of MPC level has been revealed. In addition, miR-29b modulates extracellular matrix proteins to regulate osteoblastogenesis [53]. By analysing available online databases, Ding et al. found unusual miR-99b-5p activity in osteoporosis [54]. miR-99b-5p targets the mRNA of the *FGFR3* gene encoding fibroblast growth factor receptor type 3, which is known to be a negative regulator of bone growth [55].

Of particular interest is miR-185. Yao et al. studied the effects of miR-185 on osteoblasts in mice during right femur fracture healing. They found that this miRNA suppresses osteoblast growth and proliferation during fracture healing by targeting the parathyroid hormone gene (*PTH*), completely inhibiting the canonical WNT signalling pathway. Moreover, miR-185 inhibitors promoted the restoration of osteoblast viability [56]. Blocking miR-185 expression enhances bone formation in osteoporosis, which may be partly due to the regulation of *BGN* gene expression and BMP/SMAD signalling. It has been shown that miR-185-5p is active as a negative regulator of osteoblast differentiation by inhibiting the DLX2 factor [57].

**Figure 1:**
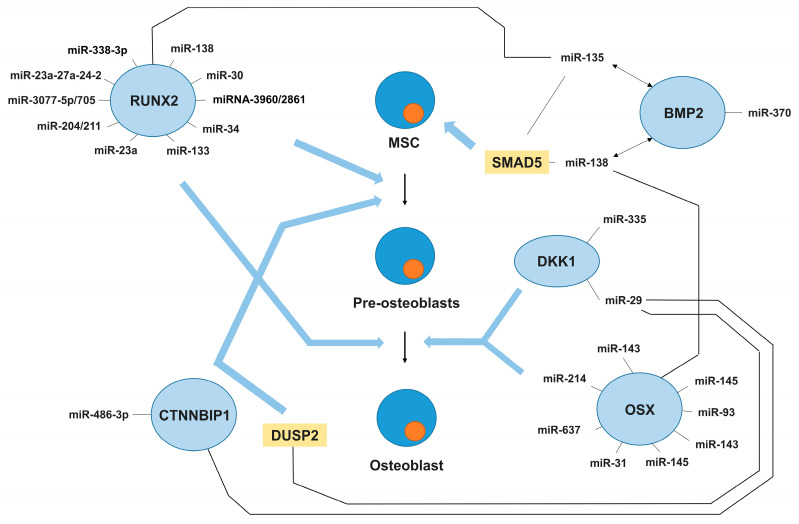
MicroRNAs regulating osteoblast differentiation.

As discussed previously, RUNX2 is a key transcription factor on which the activation of osteoblastogenesis depends. To date, a wealth of data have been accumulated on miRNAs that target RUNX2. These include miR-23a-3 [58], miR-133 [52], miR-30 [59], miR-23a∼27a∼24-2 [60], miR-34a [61], miR-138-5p [62], miR-204 [63], miR-3960/2861 [64], miR-3077-5p/705 [65], miR-338-3p [66], etc. (Figure 1). RUNX2 directs mesenchymal cells to the osteogenic lineage and inhibits adipogenic differentiation.

In addition to RUNX2, OSX, DKK1, BMP2, CTNNBIP1, and DUSP2 play important roles in osteogenesis. Figure 1 summarises the miRNAs that are involved in their regulation. Osterix (OSX), also called SP7 transcription factor, is highly conserved among vertebrates and is responsible for gene activation during the differentiation of preosteoblasts into mature osteoblasts and osteocytes [67]. The expression activity of Osterix itself is influenced by a huge number of different factors, among which are miRNAs: miR-214 [68], miR-93 [69], miR-143, miR-145 [70], miR-31 [71], and miR-637 [72]. The DKK1 protein is an inhibitor of the WNT signalling pathway and plays one of the key roles in processes related to the development and maintenance of bone tissue homeostasis. There is not much data on the role of miRNAs affecting *DKK1* gene expression, but several studies report that miR-355 [73] and miR-29 [74] are regulators of DKK1.

Bone morphogenetic protein type 2 (BMP2) plays an important role in osteoblast differentiation, which also provides a chemotactic gradient for bone marrow MSC recruitment to initiate bone regeneration. The direct relationship between miRNA dynamics and their regulation by BMP2 in osteoporosis is poorly understood; however, it is known that miR-370 and miR-138 expression levels tend to increase when BMP2 levels decrease in colorectal cancer [75]. It is known that miR-135 affects SMAD5, a key osteogenic signal transducer of BMP2, thus affecting MSC differentiation [76]. The β-catenin-interacting protein 1 (CTNNBIP1) is an inhibitor of WNT/β-catenin signalling. An inverse relationship between CTNNBIP1 levels and miR-486-3p (which targets the mRNA of the *CTNNBIP1* gene) was found. The level of this miRNA was decreased in osteoporosis [77]. Also worth mentioning is DUSP2, a member of the dual-specificity protein phosphatase subfamily. These inactivating phosphatases play an important role in osteogenic differentiation. The mRNA of the *DUSP2* gene is targeted by miR-29 [78]. Thus, a considerable amount of evidence has accumulated indicating that miRNAs are involved in the regulation of bone metabolism gene expression.

## 4. Role of microRNAs in the Regulation of Osteoclast Differentiation and Activity

Osteoclasts are cells of haematopoietic origin that are specialised in bone resorption. These cells are multinucleated (10 to 100 nuclei, 40 to 200 µm in size) and extremely mobile. Through the release of hydrolytic enzymes, osteoclasts dissolve the mineral matrix of hydroxyapatite and degrade collagen. Osteoclast formation is controlled by macrophage colony-stimulating factor, which promotes the differentiation of myeloid precursors into promonocytes, and by the receptor activator of nuclear factor kappa B ligand (RANKL), a potent cytokine that induces the differentiation of promonocytes into osteoclasts. Loss of bone mass is usually a consequence of excessively increased osteoclast activity [79]. For this reason, abnormal activation of osteoclasts can lead to decreased BMD, causing osteopenia, OP, and other bone diseases [80].

**Figure 2:**
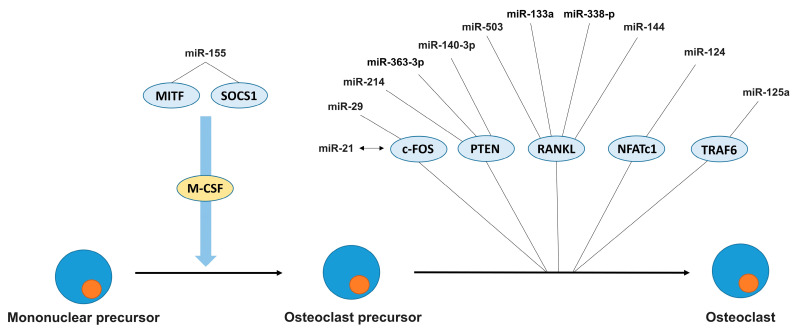
MicroRNAs regulating osteoсlast differentiation.

At first glance, there is not much data on the involvement of miRNAs in different stages of osteoclastogenesis. Nevertheless, a number of studies have confirmed the participation of certain miRNAs in the regulation of key mechanisms of osteoclastogenesis [81]. Figure 2 shows miRNAs whose role in osteoclastogenesis has been functionally confirmed. The role of other miRNAs requires further research.

In particular, miR-144-3p and miR-133a regulate the RANKL-RANK-OPG ligand-receptor system, a central component of osteoclastogenesis [82]. miR-503 also targets RANK, and there is also evidence that the inhibition of this miRNA enhances RANKL-induced osteoclastogenesis [83]. miR-124 is a specific suppressor of NFATc1, a cytoplasmic activation factor of type 1 T cells, and through it, it can regulate osteoclast differentiation, proliferation, and migration [84]. The inhibition of miR-21 biogenesis by oestrogen has been found to promote osteoclast apoptosis. The regulatory effects of miR-21 on osteoclasts are complex and involve multiple mechanisms. The work of Sugatani et al. showed that miR-21 expression is upregulated during RANKL-induced osteoclastogenesis. It was hypothesised that the activity of this miRNA in osteoclasts is associated with a positive feedback loop with the C-Fos/miR-21/PDCD4 system [85].

C-Fos increases miR-21 expression and decreases *PDCD4* gene expression, and reduced PDCD4 levels result in a reduced inhibitory effect of C-Fos, which in turn promotes osteoclastogenesis. miR-21 affects osteoclasts by also influencing the ratio of RANKL/OPG levels [86]. miR-338-3p targets the MafB transcription factor gene, promoting osteoclast differentiation [87]. Three other miRNAs are involved in the PTEN-PI3K-AKT signalling pathway, one of the key cell cycle pathways: miR-140-3p and miR-363-3p contribute to its induction, while miR-2861, in contrast, leads to its inhibition [81]. Of equal interest is miR-1270, which is overexpressed in circulating monocytes [88]. Bioinformatics analysis revealed that the mRNA of interferon regulatory factor 8 (IRF8), an osteoclastogenesis-related regulatory factor, contains a binding site for miR-1270. When miR-1270 levels are high in circulating monocytes, *IRF8* gene expression is reduced, which was confirmed in a study of postmenopausal women. Jiménez-Ortega et al. found an overexpression of miR-1270, miR-548-3p, and miR-8084 in Mexican mestizo mononuclear (N = 12) from postmenopausal women with OP [80]. Notably, miR-338-3p suppressed glucocorticoid-induced osteoclast formation by targeting RANKL. In contrast, dexamethasone stimulates osteoclast differentiation and suppresses miR-338-3p expression [89]. miR-214 promotes osteoclastogenesis by activating the PTEN/PI3K/AKT signalling pathway. It does this by increasing the expression of macrophage colony-stimulating factor (M-CSF) and receptor activator of nuclear factor-κB (RANKL) during osteoclast differentiation from bone marrow-derived monocytes. This suggests that miR-214 is essential for the formation of osteoclasts [90].

The research group of Rossi et al. at the same time showed that the expression level of miR-29b gradually decreases during the differentiation of CD14+ cells into osteoclasts [91]. However, there is evidence that contradicts these findings: as suggested by the group of Franceschetti et al., miR-29 is not a negative but a positive regulator of osteoclast maturation. According to the authors, this miRNA targets the mRNA of nuclear factor 1 type A (NFI-A) genes, inhibiting the differentiation of monocytes into the macrophage lineage. Given that low levels of NFI-A promote the active functioning of the M-CSFR receptor, which is crucial for osteoclast differentiation, it is reasonable to speak of a positive effect of this miRNA on resorption function. The study showed that both primary bone marrow cells and the RAW264.7 monocytic cell line produced smaller osteoclasts upon miR-29a knockout without a loss of cell viability. Thus, there are conflicting data on the function of miR-29 in osteoclastogenesis, which is particularly interesting given the significant functional role of this miRNA in osteoblastogenesis as well [92].

## 5. MicroRNA as a Marker of Osteoporosis Treatment Efficacy

One interesting area of research in the field of miRNAs involves their role in the regulation of osteoblast and osteoclast differentiation under normal and osteoporotic conditions. Researchers are seeking to identify miRNAs that could serve as biomarkers for the effectiveness of treatments, such as bisphosphonates and monoclonal antibodies, in treating the disease (Table 1).

In particular, studies have shown that levels of specific miRNAs change during treatment with bisphosphonates and teriparatide in animal models of postmenopausal osteoporosis. Kocijan et al. performed NGS sequencing of RNA in bone cells and peripheral blood cells. Ovariectomised rats were divided into subgroups depending on the antiresorptive therapy drugs used and found that the levels of 10 miRNAs were significantly altered by zoledronate treatment and 46 by teriparatide treatment. In particular, miR-133 was reduced with zoledronic acid therapy, and this miRNA is known to target RUNX2. At the same time, levels of miR-203a and miR-20a-5p were also reduced on teriparatide therapy. They are thought to regulate the activity of osteoblast differentiation transcription factors, including RUNX2, BMP2, and PPAR, and thus osteoblast maturation and bone formation [93].

miRNA studies on the background of osteoporosis therapy with denosumab have not been neglected. Two years of monoclonal drug treatment led to an increase in the levels of seven miRNAs (miR-101-3p, miR-191-5p, miR-26b-5p, miR-32-5p, miR-4508, miR-454-3P, and miR-584-5p). Among these, miR-454-3p and miR-191-5p were highly expressed in monocytes, while miR-26b-5p was also increased. Therefore, a change in the expression of these miRNAs may be a marker for an increase in bone mineral density during denosumab treatment [94].

Glucocorticoid-induced osteoporosis is one of the most frequent forms of secondary OP. Low miR-199a-5p levels are associated with the glucocorticoid-mediated suppression of osteogenesis. An overexpression of miR-199a-5p increases the inhibitory effect of dexamethasone on osteoblast proliferation. It was found that miR-199a-5p decreases the expression of *FZD4* and *WNT2* genes, which further confirmed the important role of this miRNA in WNT signalling and the relationship with glucocorticoid-induced osteoporosis [95].

These studies do not allow us to directly link epigenetics and osteoporosis to classical therapy, but they may allow us to use different miRNAs as markers for treatment efficacy and gain a better understanding of the role of epigenetic modulators in osteoporosis pathogenesis.

## 6. Circulating microRNAs for Predicting Osteoporosis Risk

To date, a wide range of studies on miRNAs have created a scientific basis for developments in the field of diagnostics of various diseases using customised panels of miRNAs, in particular, based on circulating miRNAs. The prognostic power of the developed panels, where serum miRNAs are selected as biomarkers, makes it possible to identify individuals at high risk of fractures in OP [96].

Studies demonstrate that the level of some miRNAs significantly increases, while the level of other miRNAs in serum decreases in patients with osteoporosis compared to the control group without osteoporosis [97,98]. Several studies have found that the dynamics of certain circulating miRNAs are associated in patients with osteoporotic fractures: these are miR-21, miR-23a, miR-24, miR-93, miR-100, miR-122a, miR-124a, miR-125b, miR-148a [99], miR-10a-5p, miR-10b-5p, and miR-22-3p [100], which are significantly elevated in the serum of patients with osteoporosis. Median relative expression levels of miR-187 are 5.3-fold higher in the fracture-free group. In contrast, miR-518f is expressed 8.6-fold higher in the bone of patients with osteoporosis [12]. A collaboration of research groups from the US and Austria found that miR-382-3p and miR-550a-5p are associated with fractures due to bone fragility in postmenopausal women with type 2 diabetes. miR-382-3p significantly enhances osteogenic differentiation, whereas miR-550a-5p inhibits this process. Both miRNAs impair adipogenic differentiation [101].

Zarecki et al. conducted a study on miRNAs in postmenopausal women in three groups: those with vertebral fractures, those with low bone mineral density (BMD) without fractures, and a control group. The researchers found that the levels of certain miRNAs, including miR-375, miR-532-3p, miR-19b-3p, miR-152-3p, miR-23a-3p, and miR-335-5p, in patients with fractures and low BMD were significantly higher than in the other two groups. One strength of this study was the comprehensive approach to statistical analysis. The researchers used principal component analysis to analyse the data and calculated the power of the effects they studied. This allowed them to draw more accurate conclusions about the relationship between miRNAs and bone health. miR-19b-3p affects the oestrogen signalling pathway by inhibiting the oestrogen α receptor, which may play a role in bone loss. It is noteworthy that miR-375, which was previously identified by Gayor and Weber, has been shown to inhibit osteoblastogenesis. This consistency of findings supports the results and enhances the value of the scientific work in the search for epigenetic markers of fractures in osteoporosis [102]. Ladang et al. conducted a pilot study to evaluate the prognostic value of OsteomiR (a registered system that measures fracture risk based on miR-335-5p, miR-152-3p, miR-127-3p, miR-320a, miR-144-5p, miR-582-5p, miR-17-5p, miR375, miR-188-5p, and miR141-3p) using a combined score based on a multivariate model. They derived a predictive model with maximum sensitivity (76%) and specificity (68%). OsteomiR levels were higher in patients with osteopenia and osteoporosis compared to patients with normal T-scores. In addition, OsteomiR scores predicted more fracture events than the recommended ‘need for treatment’ thresholds based on the 10-year FRAX probability. Thus, miRNAs reflect disturbances in bone homeostasis several years before a fracture occurs [103]. The study team enrolled 50 postmenopausal women with osteoporosis (with or without fractures due to fragility) and 50 women without osteoporosis in 2021. The paper also evaluated the prognostic efficacy of OsteomiR using multiple logistic regression and receiver-operator curve (AUC) analysis. The authors summarised that the OsteomiR signature consists of four miRNA clusters that provide good performance for the diagnosis of OP in postmenopausal women, among which miR-375 (high levels associated with the risk of OP (OR = 2.45)) and miR-203a (high levels associated with the risk of fracture OR = 2.78) contributed the most [96].

Wu et al. performed a meta-analysis and summarised the results of all major studies investigating miRNAs in osteoporosis. According to the authors, there are only six miRNAs that are noteworthy, envisaging their diagnostic power as biomarkers, which include miR-23b-3p, miR-140-3p, miR-155-5p, miR-208a-3p, miR-300, and miR-637 [81]. The authors concluded that these miRNAs are preferred candidates for diagnostic panels.

However, there is an urgent need to validate these arguments to establish a solid foundation and consensus on the applicability of these miRNAs in practice due to the small sample size in most studies and little overlap in identified miRNAs across studies.

## 7. Development of Gene Therapeutic Agents Based on microRNAs

miRNAs involved in various parts of gene activity regulation represent a new and potentially powerful class of candidates for targeting drugs for personalised therapy. Although information about miRNAs continues to expand, we still do not fully understand how effective miRNA-based constructs will be in targeting gene activity. Various research groups around the world and pharmaceutical companies are researching and searching for the most effective miRNA delivery system and developing miRNA-based antisense therapy technologies. Osteoporosis is a heterogeneous polygenic disease, the pathogenesis of which is determined by different mechanisms and a large number of genes and epigenetic regulatory factors, which complicates the search for reliable gene-therapeutic constructs based on any specific miRNAs.

Nevertheless, to date, there are at least several miRNA-based antisense constructs that have been successfully conducted and tested in animal models or human cell lines and confirmed to be effective in the treatment of osteoporosis. In 2013, Suh et al. developed a non-toxic, arginine-rich low-molecular-weight protamine (LMWP) in which a synthetic double-stranded miR-29b mimic was transduced directly into MSCs, which, according to the results of the experiment, resulted in the stimulation of MSCs towards osteogenic differentiation through a significant decrease in the expression of the *HDAC4* (histone deacetylase 4), *CTNNBIP1* (beta-catenin interacting protein 1), and *DUSP2* (protein phosphatase 2) genes. The authors of the study summarised that LMWP is a potent non-toxic cell-penetrating carrier for miR-29b, and that the effect of bone tissue mineralisation under the influence of the LMWP/miR-29b complex in human MSCs is potentially applicable as a tool to stimulate stem cell differentiation [104].

A little later, in 2015, a group of Chinese researchers developed a novel miRNA delivery system based on MS2 bacteriophage virus-like particles (MS2 VLP) and used this system to transport miR-146a into human peripheral blood mononuclear cells (PBMCs) and demonstrated the inhibition of osteoclastogenesis in progenitors. The system demonstrated high delivery efficiency and potential applicability [105]. In the same year, a paper was published by a group of researchers from the USA, who reported the successful application of photoactivated conjugates on nanoparticles containing miR-148b, which allowed them to repair critical defects of the skull vault in mice. The authors assure that this technology can offer an effective way to modulate osteogenic differentiation, which can be used with other miRNAs that regulate regeneration and wound healing processes.

A little later, in 2019, another study was published, in which a team of Chinese scientists designed an adeno-associated virus (AAV)-conjugated genetic construct targeting miR-214 (AAV-anti-miR-214), which plays an important role in blocking osteoblastogenesis. As a result, the authors were able to confirm that AAV-anti-miR-214 indeed stimulates osteoblast activity and reduces osteoclast activity, effectively preventing femoral head collapse in an osteonecrosis model. Given that osteonecrosis is a frequent side effect of bisphosphonate therapy, the results of this study are of great value for developments to improve existing therapies [106].

Thus, due to technological advances and the identification of effective miRNA delivery methods, miRNA-based therapies hold great promise. We hope that with the growing number of such studies and increasing interest in this field, biotechnology companies will have new prospects for developing targeted therapies for osteoporosis.

## 8. Conclusions

One of the most acute problems in the health care system is the lack of comprehensive early diagnosis and prevention of osteoporosis. Since the disease is latent for a long time, and patients are usually referred to non-targeted specialists at first contact, there is a problem in the untimely diagnosis of osteoporosis. A great number of studies of molecular genetics of osteoporosis have been carried out all over the world, and methods of early DNA-diagnostics have been patented; however, the results of these studies mostly do not find practical applications. Great hopes are pinned on osteoporosis epigenetics research, the results of which show high potential for the development of targeted therapy and early diagnosis. The results of studies have shown the diversity and comprehensive involvement of miRNAs in all links of bone metabolism, bone remodelling, and the formation of osteoporosis risk. However, at the same time, one cannot but notice some differences in the identified miRNAs in different studies, especially in case–control studies, in which miRNAs are studied as biomarkers of fractures and low BMD.

This probably indicates the presence of a population factor, as well as the specific character of miRNA expression depending on age, sex, and severity of the disease course. In addition, miRNA expression and changes in their patterns may be influenced by changes in miRNA binding sites in the mRNA of target genes [107]. Given that miRNA activity depends on a variety of exogenous and endogenous factors, including complex gene networks of gene interactions, the involvement of miRNA binding site polymorphism in the efficiency of interactions between mRNA and miRNA, the final miRNA pattern determining the risk of disease development may be extremely dynamic and difficult to reproduce, taking into account all of the above-mentioned reasons.

Notably, some miRNAs are involved in both osteoclastogenesis and osteoblastogenesis, and their regulation is complex and linked to many regulatory factors through a double feedback principle. On the other hand, some miRNAs are specific regulators of individual genes or are associated with osteoporosis, characterising them as potential biomarkers suitable for the development of targeted therapies or diagnosis at an early stage of disease progression. Considering this, studying patients with an osteoporotic phenotype by analysing specific miRNAs in osteoclast and osteoblast precursors could lead to a more precise identification of markers that hold significant diagnostic relevance for practical medicine. It has become apparent that miRNAs play a significant role in osteoporosis. The systematic identification and understanding of the complex interactions between miRNAs and their target genes are essential for understanding the mechanisms underlying osteoporosis. miRNAs are epigenetic regulators that indirectly affect the risk of developing osteoporosis, but their precise role in the pathogenesis of the disease remains unclear. Due to the dynamism of miRNAs and the variability in their expression levels, it is challenging to systematically classify them according to their importance and functional involvement. This makes it difficult to develop methods for early diagnosis and targeted treatment of osteoporosis.

## Figures and Tables

**Table 1:** The microRNAs whose levels change during therapy with different groups of drugs.

Medicament	miRNA	Regulated Genes
denosumab	miR-101-3p	*CCND2*, *GSK3B*, *JUN*, *FZD6*, *TBL1Xr1*, *VANGL1*, *SMAD7*, *TGFBr2*, *SMAD5*, *SP1*, *FOSL2*, *IGF1r*, *FYN*
	miR-191-5p	*CCND2*, *GSK3B*, *JUN*, *TBL1Xr1*, *VANGL1*, *SMAD7*, *TGFBr2*, *SMAD5*, *SP1*, *FOSL2*, *IGF1r*, *FYN*
	miR-26b-5p	*CCND2*, *GSK3B*, *JUN*, *FZD6*, *TBL1Xr1*, *VANGL1*, *SMAD7*, *TGFBr2*, *SMAD5*, *SP1*, *FOSL2*, *IGF1r*
	miR-32-5p	*CCND2*, *GSK3B*, *JUN*, *FZD6*, *TBL1Xr1*, *VANGL1*, *SMAD7*, *SMAD5*, *SP1*
	miR-4508	*IGF1r*
	miR-454-3p	*CCND2*, *FZD6*, *TBL1Xr1*, *VANGL1*, *TGFBr2*, *SMAD5*, *SP1*, *FOSL2*, *IGF1r*
	miR-584-5p	*FYN*
teriparatide	miR-203a	*RUNX2*, *BMP2*, *PPAR*
	miR-20a-5p	
zoledronate	miR-133	*RUNX2*
dexamethasone	miR-199a-5p	*FZD4*, *WNT2*

## References

[1] Salari N., Ghasemi H., Mohammadi L., Behzadi M.H., Rabieenia E., Shohaimi S., Mohammadi M. (2021). The global prevalence of osteoporosis in the world: A comprehensive systematic review and meta-analysis. J. Orthop. Surg. Res..

[2] Lesnyak O.M., Baranova I.A., Belova K.Y. (2018). Osteoporosis in Russian Federation: Epidemiology, socio-medical and economical aspects (review). Traumatol. Orthop. Russ..

[3] Belaya Z., Rozhinskaya L., Dedov I., Drapkina O., Fadeev V., Golounina O., Lesnyak O., Mamedova E., Melnichenko G., Petraikin A. (2023). A summary of the Russian clinical guidelines on the diagnosis and treatment of osteoporosis. Osteoporos. Int..

[4] Liang B., Burley G., Lin S., Shi Y.C. (2022). Osteoporosis pathogenesis and treatment: Existing and emerging avenues. Cell. Mol. Biol. Lett..

[5] Chen Y., Sun Y., Xue X., Ma H. (2023). Comprehensive analysis of epigenetics mechanisms in osteoporosis. Front. Genet..

[6] Yalaev B.I., Tyurin A.V., Mirgalieva R.I., Khusnutdinova E.K., Khusainova R.I. (2021). Investigating the role of osteoprotegerin gene polymorphic variants in osteoporosis. Russ. Open Med. J..

[7] Letarouilly J.G., Broux O., Clabaut A. (2019). New insights into the epigenetics of osteoporosis. Genomics.

[8] Xu F., Li W., Yang X., Na L., Chen L., Liu G. (2021). The Roles of Epigenetics Regulation in Bone Metabolism and Osteoporosis. Front. Cell Dev. Biol..

[9] Yalaev B.I., Khusainova R.I. (2023). Epigenetic regulation of bone remodeling and its role in the pathogenesis of primary osteoporosis. Vavilovskii Zhurnal Genet. Sel..

[10] Reppe S., Lien T.G., Hsu Y.H., Gautvik V.T., Olstad O.K., Yu R., Bakke H.G., Lyle R., Kringen M.K., Glad I.K. (2017). Distinct DNA methylation profiles in bone and blood of osteoporotic and healthy postmenopausal women. Epigenetics.

[11] Cheishvili D., Parashar S., Mahmood N., Arakelian A., Kremer R., Goltzman D., Szyf M., Rabbani S.A. (2018). Identification of an Epigenetic Signature of Osteoporosis in Blood DNA of Postmenopausal Women. J. Bone Miner. Res..

[12] Garmilla-Ezquerra P., Sañudo C., Delgado-Calle J., Pérez-Nuñez M.I., Sumillera M., Riancho J.A. (2015). Analysis of the bone MicroRNome in osteoporotic fractures. Calcif. Tissue Int..

[13] Yang Y.Q., Yu X.H., Bo L., Lei S.F., Deng F.Y. (2022). Genetic Risk for Osteoporosis and the Benefit of Adherence to Healthy Lifestyles. Int. J. Public Health.

[14] Bolamperti S., Villa I., Rubinacci A. (2022). Bone remodeling: An operational process ensuring survival and bone mechanical competence. Bone Res..

[15] Jiang M., Liu R., Liu L., Kot A., Liu X., Xiao W., Jia J., Li Y., Lam K.S., Yao W. (2020). Identification of osteogenic progenitor cell-targeted peptides that augment bone formation. Nat. Commun..

[16] Kylmäoja E., Nakamura M., Turunen S., Patlaka C., Andersson G., Lehenkari P., Tuukkanen J. (2018). Peripheral blood monocytes show increased osteoclast differentiation potential compared to bone marrow monocytes. Heliyon.

[17] Lozano C., Duroux-Richard I., Firat H., Schordan E., Apparailly F. (2019). MicroRNAs: Key regulators to understand osteoclast differentiation?. Front. Immunol..

[18] Yang T.L., Shen H., Liu A., Dong S.S., Zhang L., Deng F.Y., Zhao Q., Deng H.W. (2020). A road map for understanding molecular and genetic determinants of osteoporosis. Nat. Rev. Endocrinol..

[19] Peng S., Cao L., He S., Zhong Y., Ma H., Zhang Y., Shuai C. (2018). An Overview of Long Noncoding RNAs Involved in Bone Regeneration from Mesenchymal Stem Cells. Stem Cells Int..

[20] Yin C., Tian Y., Yu Y., Wang H., Wu Z., Huang Z., Zhang Y., Li D., Yang C., Wang X. (2019). A novel long noncoding RNA AK016739 inhibits osteoblast differentiation and bone formation. J. Cell. Physiol..

[21] Yuan H., Xu X., Feng X., Zhu E., Zhou J., Wang G., Tian L., Wang B. (2019). A novel long noncoding RNA PGC1β-OT1 regulates adipocyte and osteoblast differentiation through antagonizing miR-148a-3p. Cell Death Differ..

[22] Zhang Y., Xue W., Li X., Zhang J., Chen S., Zhang J.L., Yang L., Chen L.L. (2016). The Biogenesis of Nascent Circular RNAs. Cell Rep..

[23] Dou C., Cao Z., Yang B., Ding N., Hou T., Luo F., Kang F., Li J., Yang X., Jiang H. (2016). Changing expression profiles of lncRNAs, mRNAs, circRNAs and miRNAs during osteoclastogenesis. Sci. Rep..

[24] Ma B., Wang S., Wu W., Shan P., Chen Y., Meng J., Xing L., Yun J., Hao L., Wang X. (2023). Mechanisms of circRNA/lncRNA-miRNA interactions and applications in disease and drug research. Biomed. Pharmacother..

[25] Annese T., Tamma R., De Giorgis M., Ribatti D. (2020). microRNAs Biogenesis, Functions and Role in Tumor Angiogenesis. Front. Oncol..

[26] Creamer K.M., Partridge J.F. (2011). RITS-connecting transcription, RNA interference, and heterochromatin assembly in fission yeast. Wiley Interdiscip. Rev. RNA.

[27] Yu L., Li W., Yang P., Zhang W., Tao H., Ge G., Yang H., Bai J., Wang H., Geng D. (2022). Osteoblastic microRNAs in skeletal diseases: Biological functions and therapeutic implications. Eng. Regen..

[28] Hagh M.F., Noruzinia M., Mortazavi Y., Soleimani M., Kaviani S., Abroun S., Fard A.D., Maymand M.M. (2015). Different Methylation Patterns of RUNX2, OSX, DLX5and BSP in Osteoblastic Differentiation ofMesenchymal Stem Cells. Cell J..

[29] Yao J., Xin R., Zhao C., Yu C. (2024). MicroRNAs in osteoblast differentiation and fracture healing: From pathogenesis to therapeutic implication. Injury.

[30] Ghayor C., Weber F.E. (2016). Epigenetic regulation of bone remodeling and its impacts in osteoporosis. Int. J. Mol. Sci..

[31] Chen C., Cheng P., Xie H., Zhou H.D., Wu X.P., Liao E.Y., Luo X.H. (2014). MiR-503 regulates osteoclastogenesis via targeting RANK. J. Bone Miner. Res..

[32] Hu H., He X., Zhang Y., Wu R., Chen J., Lin Y., Shen B. (2020). MicroRNA Alterations for Diagnosis, Prognosis, and Treatment of Osteoporosis: A Comprehensive Review and Computational Functional Survey. Front. Genet..

[33] Grebennikova T.A., Belaya Z.E., Rozhinskaya L.Y., Mel’nichenko G.A., Dedov I.I. (2015). Epigenetic aspects of osteoporosis. Vestn. Ross. Akad. Meditsinskikh Nauk.

[34] Belaya Z., Grebennikova T., Melnichenko G., Nikitin A., Solodovnikov A., Brovkina O., Grigoriev A., Rozhinskaya L., Lutsenko A., Dedov I. (2018). Effects of active acromegaly on bone mRNA and microRNA expression patterns. Eur. J. Endocrinol..

[35] Palumbo C., Ferretti M. (2021). The osteocyte: From “prisoner” to “orchestrator”. J. Funct. Morphol. Kinesiol..

[36] Schaffler M.B., Cheung W.Y., Majeska R., Kennedy O. (2014). Osteocytes: Master orchestrators of bone. Calcif. Tissue Int..

[37] Li H., Li T., Fan J., Li T., Fan L., Wang S., Weng X., Han Q., Zhao R.C. (2015). MIR-216a rescues dexamethasone suppression of osteogenesis, promotes osteoblast differentiation and enhances bone formation, by regulating c-Cbl-mediated PI3K/AKT pathway. Cell Death Differ..

[38] Peng S., Gao D., Gao C., Wei P., Niu M., Shuai C. (2016). MicroRNAs regulate signaling pathways in osteogenic differentiation of mesenchymal stem cells (Review). Mol. Med. Rep..

[39] van Leeuwen J.P.T.M., van der Eerden B.C.J., van de Peppel J., Stein G.S., Lian J.B. (2013). Osteoblast Biology. Osteoporos.

[40] Hu L., Yin C., Zhao F., Ali A., Ma J., Qian A. (2018). Mesenchymal stem cells: Cell fate decision to osteoblast or adipocyte and application in osteoporosis treatment. Int. J. Mol. Sci..

[41] Chan W.C.W., Tan Z., To M.K.T., Chan D. (2021). Regulation and role of transcription factors in osteogenesis. Int. J. Mol. Sci..

[42] Houschyar K.S., Tapking C., Borrelli M.R., Popp D., Duscher D., Maan Z.N., Chelliah M.P., Li J., Harati K., Wallner C. (2019). Wnt Pathway in Bone Repair and Regeneration—What Do We Know So Far. Front. Cell Dev. Biol..

[43] Tang X., Lin J., Wang G., Lu J. (2017). MicroRNA-433-3p promotes osteoblast differentiation through targeting DKK1 expression. PLoS ONE.

[44] Duan L., Zhao H., Xiong Y., Tang X., Yang Y., Hu Z., Li C., Chen S., Yu X. (2018). MiR-16-2∗ interferes with WNT5A to regulate osteogenesis of mesenchymal stem cells. Cell. Physiol. Biochem..

[45] Cheng P., Chen C., He H.B., Hu R., Zhou H.D., Xie H., Zhu W., Dai R.C., Wu X.P., Liao E.Y. (2013). MiR-148a regulates osteoclastogenesis by targeting V-maf musculoaponeurotic fibrosarcoma oncogene homolog B. J. Bone Miner. Res..

[46] Gao J., Yang T., Han J., Yan K., Qiu X., Zhou Y., Fan Q., Ma B. (2011). MicroRNA expression during osteogenic differentiation of human multipotent mesenchymal stromal cells from Bone Marrow. J. Cell. Biochem..

[47] Bedene A., Mencej Bedrač S., Ješe L., Marc J., Vrtačnik P., Preželj J., Kocjan T., Kranjc T., Ostanek B. (2016). MiR-148a the epigenetic regulator of bone homeostasis is increased in plasma of osteoporotic postmenopausal women. Wien. Klin. Wochenschr..

[48] Mäkitie R.E., Hackl M., Niinimäki R., Kakko S., Grillari J., Mäkitie O. (2018). Altered MicroRNA Profile in Osteoporosis Caused by Impaired WNT Signaling. J. Clin. Endocrinol. Metab..

[49] Weilner S., Schraml E., Wieser M., Messner P., Schneider K., Wassermann K., Micutkova L., Fortschegger K., Maier A.B., Westendorp R. (2016). Secreted microvesicular miR-31 inhibits osteogenic differentiation of mesenchymal stem cells. Aging Cell.

[50] Mizoguchi F., Murakami Y., Saito T., Miyasaka N., Kohsaka H. (2013). MiR-31 controls osteoclast formation and bone resorption by targeting RhoA. Arthritis Res. Ther..

[51] Ramírez-Salazar E.G., Carrillo-Patiño S., Hidalgo-Bravo A., Rivera-Paredez B., Quiterio M., Ramírez-Palacios P., Patiño N., Valdés-Flores M., Salmerón J., Velázquez-Cruz R. (2018). Serum miRNAs miR-140-3p and miR-23b-3p as potential biomarkers for osteoporosis and osteoporotic fracture in postmenopausal Mexican-Mestizo women. Gene.

[52] Tang P., Xiong Q., Ge W., Zhang L. (2014). The role of MicroRNAs in osteoclasts and osteoporosis. RNA Biol..

[53] Lehmann T.P., Guderska U., Kałek K., Marzec M., Urbanek A., Czernikiewicz A., Sąsiadek M., Karpiński P., Pławski A., Głowacki M. (2022). The regulation of collagen processing by miRNAs in disease and possible implications for bone turnover. Int. J. Mol. Sci..

[54] Ding M., Liu B., Chen X., Ouyang Z., Peng D., Zhou Y. (2021). MiR-99b-5p suppressed proliferation of human osteoblasts by targeting FGFR3 in osteoporosis. Hum. Cell.

[55] Wen X., Li X., Tang Y., Tang J., Zhou S., Xie Y., Guo J., Yang J., Du X., Su N. (2016). Chondrocyte FGFR3 regulates bone mass by inhibiting osteogenesis. J. Biol. Chem..

[56] Yao C.J., Lv Y., Zhang C.J., Jin J.X., Xu L.H., Jiang J., Geng B., Li H., Xia Y.A.Y., Wu M. (2018). MicroRNA-185 inhibits the growth and proliferation of osteoblasts in fracture healing by targeting PTH gene through down-regulating Wnt/β-catenin axis: In an animal experiment. Biochem. Biophys. Res. Commun..

[57] Cui Q., Xing J., Yu M., Wang Y., Xu J., Gu Y., Nan X., Ma W., Liu H., Zhao H. (2019). Mmu-miR-185 depletion promotes osteogenic differentiation and suppresses bone loss in osteoporosis through the Bgn-mediated BMP/Smad pathway. Cell Death Dis..

[58] Yang J.X., Xie P., Li Y.S., Wen T., Yang X.C. (2020). Osteoclast-derived miR-23a-5p-containing exosomes inhibit osteogenic differentiation by regulating Runx2. Cell. Signal..

[59] Zhang L., Li G., Wang K., Wang Y., Dong J., Wang H., Xu L., Shi F., Cao X., Hu Z. (2020). MiR-30 family members inhibit osteoblast differentiation by suppressing Runx2 under unloading conditions in MC3T3-E1 cells. Biochem. Biophys. Res. Commun..

[60] Hassan M.Q., Gordon J.A.R., Beloti M.M., Croce C.M., Van Wijnen A.J., Stein J.L., Stein G.S., Lian J.B. (2010). A network connecting Runx2, SATB2, and the miR-23a∼27a∼24-2 cluster regulates the osteoblast differentiation program. Proc. Natl. Acad. Sci. USA.

[61] Zha X., Sun B., Zhang R., Li C., Yan Z., Chen J. (2018). Regulatory effect of microRNA-34a on osteogenesis and angiogenesis in glucocorticoid-induced osteonecrosis of the femoral head. J. Orthop. Res..

[62] Yan F., Huo Q., Zhang W., Wu T., Dilimulati D., Shi L. (2022). MiR-138-5p targets RUNX2 to inhibit osteogenic differentiation of aortic valve interstitial cells via Wnt/β-catenin signaling pathway. BMC Cardiovasc. Disord..

[63] Huang J., Zhao L., Xing L., Chen D. (2010). MicroRNA-204 regulates Runx2 protein expression and mesenchymal progenitor cell differentiation. Stem Cells.

[64] Koenen R.R., Aikawa E. (2018). Editorial: Extracellular Vesicle-Mediated Processes in Cardiovascular Diseases. Front. Cardiovasc. Med..

[65] Liao L., Yang X., Su X., Hu C., Zhu X., Yang N., Chen X., Shi S., Shi S., Jin Y. (2013). Redundant miR-3077-5p and miR-705 mediate the shift of mesenchymal stem cell lineage commitment to adipocyte in osteoporosis bone marrow. Cell Death Dis..

[66] Lin C., Yu S., Jin R., Xiao Y., Pan M., Pei F., Zhu X., Huang H., Zhang Z., Chen S. (2019). Circulating miR-338 Cluster activities on osteoblast differentiation: Potential diagnostic and therapeutic targets for postmenopausal osteoporosis. Theranostics.

[67] Sinha K.M., Zhou X. (2013). Genetic and molecular control of osterix in skeletal formation. J. Cell. Biochem..

[68] Shi K., Lu J., Zhao Y., Wang L., Li J., Qi B., Li H., Ma C. (2013). MicroRNA-214 suppresses osteogenic differentiation of C2C12 myoblast cells by targeting Osterix. Bone.

[69] Yang L., Cheng P., Chen C., He H.B., Xie G.Q., Zhou H.D., Xie H., Wu X.P., Luo X.H. (2012). miR-93/Sp7 function loop mediates osteoblast mineralization. J. Bone Miner. Res..

[70] Wang C., Liao H., Cao Z. (2016). Role of osterix and microRNAs in bone formation and tooth development. Med. Sci. Monit..

[71] Baglìo S.R., Devescovi V., Granchi D., Baldini N. (2013). MicroRNA expression profiling of human bone marrow mesenchymal stem cells during osteogenic differentiation reveals Osterix regulation by miR-31. Gene.

[72] Zhang J.F., Fu W.M., He M.L., Wang H., Wang W.M., Yu S.C., Bian X.W., Zhou J., Lin M.C.M., Lu G. (2011). MiR-637 maintains the balance between adipocytes and osteoblasts by directly targeting Osterix. Mol. Biol. Cell.

[73] Zhang J., Tu Q., Bonewald L.F., He X., Stein G., Lian J., Chen J. (2011). Effects of miR-335-5p in modulating osteogenic differentiation by specifically downregulating Wnt antagonist DKK1. J. Bone Miner. Res..

[74] Wu Y., Li B., Deng D., Zhou H., Liu M., Ai H., Xin Y., Hua W., Zhao L., Li L. (2024). Circ_0036490 and DKK1 competitively bind miR-29a to promote lipopolysaccharides-induced human gingival fibroblasts injury. Autoimmunity.

[75] Piechowska A., Kruszniewska-Rajs C., Kimsa-Dudek M., Kołomańska M., Strzałka-Mrozik B., Gola J., Głuszek S. (2022). The role of miR-370 and miR-138 in the regulation of BMP2 suppressor gene expression in colorectal cancer: Preliminary studies. J. Cancer Res. Clin. Oncol..

[76] Li Z., Hassan M.Q., Volinia S., Van Wijnen A.J., Stein J.L., Croce C.M., Lian J.B., Stein G.S. (2008). A microRNA signature for a BMP2-induced osteoblast lineage commitment program. Proc. Natl. Acad. Sci. USA.

[77] Zhang Z., Jiang W., Hu M., Gao R., Zhou X. (2021). MiR-486–3p promotes osteogenic differentiation of BMSC by targeting CTNNBIP1 and activating the Wnt/β-catenin pathway. Biochem. Biophys. Res. Commun..

[78] Li Z., Hassan M.Q., Jafferji M., Aqeilan R.I., Garzon R., Croce C.M., van Wijnen A.J., Stein J.L., Stein G.S., Lian J.B. (2009). Biological functions of miR-29b contribute to positive regulation of osteoblast differentiation. J. Biol. Chem..

[79] Arnett T.R. (2020). Osteoclast biology. Marcus Feldman’s Osteoporos.

[80] Jiménez-Ortega R.F., Ramírez-Salazar E.G., Parra-Torres A.Y., Muñoz-Montero S.A., Rangel-Escareňo C., Salido-Guadarrama I., Rodriguez-Dorantes M., Quiterio M., Salmerón J., Velázquez-Cruz R. (2017). Identification of microRNAs in human circulating monocytes of postmenopausal osteoporotic Mexican-Mestizo women: A pilot study. Exp. Ther. Med..

[81] Wu Y.Z., Huang H.T., Cheng T.L., Lu Y.M., Lin S.Y., Ho C.J., Lee T.C., Hsu C.H., Huang P.J., Huang H.H. (2021). Application of microrna in human osteoporosis and fragility fracture: A systemic review of literatures. Int. J. Mol. Sci..

[82] Li Z., Zhang W., Huang Y. (2018). MiRNA-133a is involved in the regulation of postmenopausal osteoporosis through promoting osteoclast differentiation. Acta Biochim. Biophys. Sin..

[83] Huang M.Z., Zhuang Y., Ning X., Zhang H., Shen Z.M., Shang X.W. (2020). Artesunate inhibits osteoclastogenesis through the miR-503/RANK axis. Biosci Rep..

[84] Lee Y., Kim H.J., Park C.K., Kim Y.G., Lee H.J., Kim J.Y., Kim H.H. (2013). MicroRNA-124 regulates osteoclast differentiation. Bone.

[85] Sugatani T., Vacher J., Hruska K.A. (2011). A microRNA expression signature of osteoclastogenesis. Blood.

[86] Chen C., Liu Y.M., Fu B.L., Xu L.L., Wang B. (2021). MicroRNA-21: An Emerging Player in Bone Diseases. Front. Pharmacol..

[87] Sugatani T., Hruska K.A. (2013). Down-regulation of miR-21 biogenesis by estrogen action contributes to osteoclastic apoptosis. J. Cell. Biochem..

[88] Ramírez-Salazar E.G., Almeraya E.V., López-Perez T.V., Jiménez-Salas Z., Patiño N., Velázquez-Cruz R. (2022). MicroRNA-1270 Inhibits Cell Proliferation, Migration, and Invasion via Targeting IRF8 in Osteoblast-like Cell Lines. Curr. Issues Mol. Biol..

[89] Zhang X.H., Geng G.L., Su B., Liang C.P., Wang F., Bao J.C. (2016). MicroRNA-338-3p inhibits glucocorticoid-induced osteoclast formation through RANKL targeting. Genet. Mol. Res..

[90] Zhao C., Sun W., Zhang P., Ling S., Li Y., Zhao D., Peng J., Wang A., Li Q., Song J. (2015). miR-214 promotes osteoclastogenesis by targeting pten/pi3k/Akt pathway. RNA Biol..

[91] Rossi M., Pitari M.R., Amodio N., Di Martino M.T., Conforti F., Leone E., Botta C., Paolino F.M., Del Giudice T., Iuliano E. (2013). miR-29b negatively regulates human osteoclastic cell differentiation and function: Implications for the treatment of multiple myeloma-related bone disease. J. Cell. Physiol..

[92] Franceschetti T., Kessler C.B., Lee S.K., Delany A.M. (2013). MiR-29 promotes murine osteoclastogenesis by regulating osteoclast commitment and migration. J. Biol. Chem..

[93] Kocijan R., Weigl M., Skalicky S., Geiger E., Ferguson J., Leinfellner G., Heimel P., Pietschmann P., Grillari J., Redl H. (2020). MicroRNA levels in bone and blood change during bisphosphonate and teriparatide therapy in an animal model of postmenopausal osteoporosis. Bone.

[94] Messner Z., Vázquez D.C., Haschka J., Grillari J., Resch H., Muschitz C., Pietschmann P., Zwerina J., Hackl M., Kocijan R. (2023). Circulating miRNAs Respond to Denosumab Treatment after 2 Years in Postmenopausal Women with Osteoporosis—The MiDeTe study. J. Clin. Endocrinol. Metab..

[95] Shi C., Huang P., Kang H., Hu B., Qi J., Jiang M., Zhou H., Guo L., Deng L. (2015). Glucocorticoid inhibits cell proliferation in differentiating osteoblasts by microRNA-199a targeting of WNT signaling. J. Mol. Endocrinol..

[96] Kerschan-Schindl K., Hackl M., Boschitsch E., Föger-Samwald U., Nägele O., Skalicky S., Weigl M., Grillari J., Pietschmann P. (2021). Diagnostic Performance of a Panel of miRNAs (OsteomiR) for Osteoporosis in a Cohort of Postmenopausal Women. Calcif. Tissue Int..

[97] Wang Y., Li L., Moore B.T., Peng X.H., Fang X., Lappe J.M., Recker R.R., Xiao P. (2012). Mir-133a in human circulating monocytes: A potential biomarker associated with postmenopausal osteoporosis. PLoS ONE.

[98] Li H., Wang Z., Fu Q., Zhang J. (2014). Plasma miRNA levels correlate with sensitivity to bone mineral density in postmenopausal osteoporosis patients. Biomarkers.

[99] Seeliger C., Karpinski K., Haug A.T., Vester H., Schmitt A., Bauer J.S., Van Griensven M. (2014). Five freely circulating miRNAs and bone tissue miRNAs are associated with osteoporotic fractures. J. Bone Miner. Res..

[100] Weilner S., Skalicky S., Salzer B., Keider V., Wagner M., Hildner F., Gabriel C., Dovjak P., Pietschmann P., Grillari-Voglauer R. (2015). Differentially circulating miRNAs after recent osteoporotic fractures can influence osteogenic differentiation. Bone.

[101] Heilmeier U., Hackl M., Skalicky S., Weilner S., Schroeder F., Vierlinger K., Patsch J.M., Baum T., Oberbauer E., Lobach I. (2016). Serum miRNA Signatures Are Indicative of Skeletal Fractures in Postmenopausal Women With and Without Type 2 Diabetes and Influence Osteogenic and Adipogenic Differentiation of Adipose Tissue–Derived Mesenchymal Stem Cells In Vitro. J. Bone Miner. Res..

[102] Zarecki P., Hackl M., Grillari J., Debono M., Eastell R. (2020). Serum microRNAs as novel biomarkers for osteoporotic vertebral fractures. Bone.

[103] Ladang A., Beaudart C., Locquet M., Reginster J.Y., Bruyère O., Cavalier E. (2020). Evaluation of a Panel of MicroRNAs that Predicts Fragility Fracture Risk: A Pilot Study. Calcif. Tissue Int..

[104] Suh J.S., Lee J.Y., Choi Y.S., Chong P.C., Park Y.J. (2013). Peptide-mediated intracellular delivery of miRNA-29b for osteogenic stem cell differentiation. Biomaterials.

[105] Yao Y., Jia T., Pan Y., Gou H., Li Y., Sun Y., Zhang R., Zhang K., Lin G., Xie J. (2015). Using a novel MicroRNA delivery system to inhibit osteoclastogenesis. Int. J. Mol. Sci..

[106] Wang C., Sun W., Ling S., Wang Y., Wang X., Meng H., Li Y., Yuan X., Li J., Liu R. (2019). AAV-Anti-miR-214 Prevents Collapse of the Femoral Head in Osteonecrosis by Regulating Osteoblast and Osteoclast Activities. Mol. Ther. Nucleic Acids.

[107] Lei S.F., Papasian C.J., Deng H.W. (2011). Polymorphisms in predicted miRNA binding sites and osteoporosis. J. Bone Miner. Res..

